# Environmental Contamination With SARS-CoV-2 in a Hospital Setting

**DOI:** 10.7759/cureus.34136

**Published:** 2023-01-24

**Authors:** Sumit Kumar, Ishtiyaq Bukhari, Zeeshan Afzal, Sophie Tucker, Robin Lucas-Evans, Asghar Dayala, Dennis Mlangeni

**Affiliations:** 1 Surgery, Peterborough City Hospital, Peterborough, GBR; 2 Surgery, Addenbrooke’s Hospital, Cambridge University Hospitals NHS Foundation Trust, Cambridge, GBR; 3 Clinical Chemistry and Immunology, Peterborough City Hospital, Peterborough, GBR; 4 Microbiology, Peterborough City Hospital, Peterborough, GBR

**Keywords:** fomites, environmental contamination, hospital environment, sars-cov-2, covid-19

## Abstract

Background

The coronavirus disease 2019 (COVID-19) pandemic is a global concern and has changed the way we practice medicine in acute hospital settings. This is particularly true with regard to patient triage, patient risk assessment, use of personal protective equipment, and environmental disinfection. Transmission of severe acute respiratory syndrome coronavirus 2 (SARS-CoV-2) is primarily through inhalation of respiratory droplets generated through talking, coughing, or sneezing. There is, however, a potential risk that respiratory droplets settling on inanimate surfaces and objects in the hospital environment could provide a reservoir for nosocomial infections in patients and pose a healthcare risk to medical staff. Indeed, there have been previous reports of healthcare-associated outbreaks in hospitals. Several authors have argued that the risk of transmission via fomites may be insignificant. It is, however, not clear what proportion of SARS-CoV-2 infections are attributable to direct contact with fomites; a few reports have indicated possible transmission via this route. Environmental contamination with SARS-CoV-2 in healthcare institutions has been shown to vary according to the function or service provided by a unit or department. Information that identifies hospital areas that have a propensity for higher environmental burden may improve the practice of infection control and environmental cleaning and decontamination in healthcare institutions. This study aimed to investigate environmental SARS-CoV-2 contamination in the clinical areas of patients with COVID-19 infection.

Methodology

We conducted a cross-sectional study performing swabbing of frequently touched surfaces, equipment, and ventilation ducts in five specific clinical areas of Peterborough City Hospital which is part of the North West Anglia NHS Foundation Trust. The five clinical areas that were chosen for swabbing were the Emergency Department (ED), Intensive Care Unit (ICU), Isolation Ward, Respiratory Ward, and a Gastroenterology Ward that was serving as a receiving ward at the height of the second COVID-19 infection wave in the United Kingdom. Surfaces to be swabbed were divided into the patient zone, doctor zone, and nursing zone. Swabs from the chosen surfaces were collected on two consecutive days. A total of 158 surface swabs were collected during the second wave of the COVID-19 pandemic. SARS-CoV-2 RNA was detected by reverse transcription polymerase chain reaction.

Results

The most contaminated clinical areas were the three receiving wards where 12% (11/96) of the swabs were positive. Inside the patient rooms, these surfaces included bed rails and controls, bedside tables, television screens, remote control units, and the room ventilation system. Outside the patient room, these surfaces included mobile computers and computer desk surfaces in the doctors’ offices. All swabs taken from the ED and ICU were found to be negative.

Conclusions

Our study confirms the potential infection risks posed by environmental contamination with the SARS-CoV-2 virus. This highlights the importance of adequate environmental cleaning for proper infection control and prevention in healthcare settings.

## Introduction

The coronavirus disease 2019 (COVID-19) pandemic is of global concern and has changed the way we practice medicine in acute hospital settings. This is particularly true with regard to patient triage, patient risk assessment, use of personal protective equipment (PPE), and environmental disinfection. Severe acute respiratory syndrome coronavirus 2 (SARS-CoV-2) is primarily transmitted through inhalation of respiratory droplets generated through talking, coughing, or sneezing. However, there is a potential risk that respiratory droplets settling on inanimate surfaces and objects in the hospital environment can provide a reservoir for nosocomial infections in patients and pose a healthcare risk to medical staff. Indeed, there have been previous reports of healthcare-associated outbreaks in hospitals. Several authors have argued that the risk of transmission via fomites may be insignificant. However, it is not clear what proportion of SARS-CoV-2 infections are attributable to direct contact with fomites, with a few reports indicating possible transmission via this route [1,2]. Some case reports have implied that SARS-CoV-2 is transmitted between people by contact with surfaces that an infected individual has sneezed or coughed on with subsequent touching of the mouth, nose, or eyes leading to the acquisition of infection [3,4].

Environmental contamination with SARS-CoV-2 in healthcare institutions has been shown to vary according to the function or service provided by a unit or department. The information that identifies hospital areas that have a propensity for higher environmental burden may inform the practice of infection control, environmental cleaning, and decontamination in healthcare institutions. Several authors have demonstrated that SARS-CoV-2 can be detected on high-contact surfaces such as computers, bed rails, and door handles [5,6].

Understanding, recognizing, and identifying a contaminated environment as a possible source of transmission is paramount for infection prevention and control in hospitals and, ultimately, for the protection of healthcare workers.

In this study, we investigated environmental contamination in clinical areas with COVID-19 patients by collecting environmental swabs from patient rooms and wider clinical areas in different hospital departments, including wards, emergency departments (EDs), and intensive care units (ICUs).

This article was previously presented as a poster at the 2022 SAGES (Society of American Gastrointestinal and Endoscopic Surgeons) Annual Scientific Meeting on March 16, 2022.

## Materials and methods

We identified five clinical areas receiving laboratory-confirmed COVID-19 patients at Peterborough City Hospital. The five clinical areas that were chosen for swabbing included the Emergency Department (ED), Intensive Care Unit (ICU), Isolation Ward, Respiratory Ward, and a Gastroenterology Ward that was serving as a receiving ward at the height of the second wave of COVID-19 in the United Kingdom. The areas selected were thought to be the most used by either patients or healthcare professionals, which was mostly based on the experience and observation of clinician-researchers who conducted the study. Surfaces to be swabbed were divided into the patient zone, doctor zone, and nursing zone (Table 1).

**Table 1:** Environmental surfaces used for swab collection.

Patient zone	Doctor zone	Nursing zone	Other zones
Bed rails	Computer keyboard	Computer keyboards	Arterial blood gas machine
Oxygen saturation probe	Telephones	Monitors	Exit doors
Ventilation grill	Table surfaces	Table surfaces	
Bedside table			
Bed control device			
Television remote control			
Television screen			
Door handle			

We also included the ED and ICU of the hospital as the majority of infected patients needing hospitalization are admitted via the ED, while those most seriously ill and needing ventilation are admitted to the ICU. Furthermore, we included the ICU because of the potential for aerosol-associated dissemination of the SARS-CoV-2 virus during ventilation. These wards are geographically distant from one another in the hospital. Environmental swabs were collected from rooms of laboratory-confirmed COVID-19 patients and clinical areas outside the isolating rooms in the same ward. A paired sampling of 96 surfaces was performed over two consecutive days.

The swabs were taken from the pre-identified surfaces/objects from the study protocol and were repeated the next day (Table 1). For the swabbing of the environment, we used culture swabs without transport medium (Ref 09-511-5017 Nerbe Plus Germany). The swabs were then sent to the local onsite microbiology laboratory for processing within 12 hours of taking the samples. The samples were tested for SARS-CoV-2 RNA using the Abbott ID NOW COVID-19 assay performed on the Abbott ID NOW instrument (Abbott Diagnostics Scarborough, USA). The principle is a rapid molecular test utilizing an isothermal nucleic acid amplification technology intended for the qualitative detection of nucleic acid from the SARS-CoV-2 viral RNA. The test uses target SARS-CoV-2 RNA primers to amplify a unique region of the RdRp segment of the virus.

## Results

A total of 158 environmental swabs were collected from the surfaces/equipment outlined in Table 1. The highest number of positive environmental swabs were observed in the Isolation ward where on day one of swabbing 3/16 (19%) and on day two of swabbing 5/16 (31%) of surfaces were positive. Thus over the two-day period, a total of 8/32 (25%) of environmental swabs from the Isolation Ward were positive. Furthermore, a single positive result was obtained on each day of environmental swabbing of 16 samples from the Gastroenterology Ward (2/32), which correlates to 6% of environmental surfaces. From the Respiratory Medicine Ward, a total of 34 samples over the two days of environmental swabbing were collected and a single positive result (1/34) was obtained, which correlates to 3% of surfaces or items. No positive results were obtained from the 34 samples from the ED, or from the 26 samples from the ICU. Overall, a total of 11/158 (7%) environmental surfaces were positive for SARS-CoV-2 RNA at any one time during the study.

In the Isolation Ward, positive samples were obtained from bedrails, ceiling ventilation grills, a bedside table, a television remote control, television screens, desk surfaces, and mobile computer keyboards (Table 2). The two positive results from the Gastroenterology Ward were obtained from the ceiling ventilation grill in the patient room and the desk surface on the nurses’ station. In the Respiratory Ward, a single positive result was obtained from the bed control device. All swabs taken from the ED and ICU were negative.

**Table 2:** Environmental surfaces with positive viral RNA detection.

Environmental surface		Day of positive viral detection
Isolation Ward		
Patient zone	Bed rails	2
	Bed control	1
	Bedside table	1
	Television screen	2
	Television remote	1
	Ceiling ventilation grill	2
Doctor zone	Office desk surface	2
Other	Mobile ward computer	2
Respiratory Medicine ward		
Patient zone	Bed control device	2
Gastroenterology Ward		
Patient zone	Ceiling ventilation grill	1
Nursing zone	Office desk surface	2

## Discussion

Since the outbreak of COVID-19, several studies have been conducted to understand different aspects of the virus and the illnesses and threat it poses. A group of authors [7] conducted an environmental study in early 2020. In that study, they collected environmental swab samples from the isolation rooms of three laboratory-confirmed COVID-19 patients with severe COVID-19 pneumonia. Two of these patients required mechanical ventilation with a closed suction system. The third patient received high-flow oxygen and was managed with non-invasive ventilation. They were able to demonstrate that environmental contamination of SARS-CoV-2 was higher in the room of the patient receiving non-invasive high-flow oxygen therapy and non-invasive ventilation. They postulated that environmental contamination of SARS-CoV-2 could facilitate viral transmission. A similar study [8] was published by authors working in Wuhan in which they investigated the environmental contamination in hospital functional zones and on hospital objects and PPE. In total, 626 environmental samples were collected. They found that the ICU that specialized in caring for patients with novel COVID-19 pneumonia, the obstetric isolation ward for patients with novel COVID-19 pneumonia, and the isolation ward for patients with novel COVID-19 pneumonia showed the highest contamination rates. Analysis of the contamination rates for hospital objects and PPE showed that frequently touched equipment such as self-service printers, desktops/keyboards, door handles, medical equipment, telephones, hand sanitizer dispensers, and gloves demonstrated significant contamination. Because environmental contamination may serve as a reservoir for the spread of infections, the authors consider data on environmental contamination with SARS-CoV-2 to be necessary to inform hospital cleaning protocols.

While a large number of studies have shown a considerable potential risk of environmental contamination with SARS-CoV-2 in healthcare settings, these findings were not confirmed by a study conducted in an infectious disease emergency unit and an infectious disease sub-intensive care ward [9]. In that study, the investigators could only isolate SARS-CoV-2 RNA from the continuous positive airway pressure helmets of two patients who required advanced respiratory care. The authors also reiterate an important finding that staff PPE and medical equipment were negative for SARS-CoV-2 RNA. There have been numerous studies on the survival of the SARS-CoV-2 virus on porous and non-porous surfaces [10-12]. According to these studies, the virus is not viable within minutes to hours on porous surfaces but can be viable for days to weeks on non-porous surfaces.

Our study confirms the findings of other investigators that there is potential for the hospital environment to be contaminated with viral SARS-CoV-2 RNA. However, these studies only demonstrate that there is a potential infection risk, as none detected viable SARS-CoV-2 virus. Attempts to culture the SARS-CoV-2 virus from the environment on Vero E6 cells were unsuccessful in one study [9]. One of the significant findings of our study was that there were no positive results from ED and ICU. The ED is the first portal of entry into our hospital and, as such, has to deal with all COVID-19 patients admitted to our hospital. This, in turn, implies there is a significant risk of environmental contamination in this area of the hospital. The results of our study, however, show that the thorough and well-established environmental cleaning and equipment decontamination protocols put in place are very effective. The other explanation for this can be that patients generally spend more time in the room on the wards compared to the ED, hence greater exposure to the equipment toward the ward equipment.

Our institution has a medium-sized ICU involved with the management of patients with severe COVID-19-related illnesses requiring oxygen support and/or mechanical ventilation. The unit is also equipped with excellent environmental cleaning and equipment decontamination protocols. This can possibly explain the negative results obtained in our study. Furthermore, during our study, the unit was less busy and there were not many patients requiring mechanical ventilation.

The general wards of our hospital manage COVID-19 patients for a much longer duration than ED or ICU, and rooms are only thoroughly decontaminated once the patient leaves the hospital. These areas, therefore, present a higher risk that an isolated environment becomes contaminated, which is reflected in the results of the swabs from these areas.

A paired sampling of 96 different surfaces represents a significant sample size; however, this could be increased to improve the power of the study. Overall, 88% of negative results are very reassuring on behalf of the hospital infection and prevention policies; however, this could be improved further by introducing either increasing the frequency of cleaning cycles or the duration of each cleaning activity.

Our study has some limitations. It is a single-center study with a limited sample size. During the COVID-19 pandemic, the availability of resources including swab kits and freely available testing machines was scarce. This was the main reason for a smaller group of patient population. Other clinical areas such as surgical theaters and clinic rooms could have been included as well but it was logistically very difficult. Moreover, comparatively, time spent by patients in theaters or clinics is much less compared to time spent in the ward, resulting in less exposure. Nevertheless, the inclusion of these areas could have potentially strengthened our study.

Overall, our study strengthens the evidence of environmental contamination of SARS-CoV-2. This highlights the importance of adequate environmental cleaning for proper infection control and prevention. Further studies are required to establish a correlation between environmental contamination with SARS-CoV-2 and infection risks by detecting viable virus particles from the hospital environment.

## Conclusions

Our study confirms the evidence of potential infection risks posed by environmental contamination with the SARS-CoV-2 virus. This highlights the importance of adequate environmental cleaning for proper infection control and prevention in healthcare settings.
